# Manganese Neurotoxicity: a Focus on Glutamate Transporters

**DOI:** 10.1186/2052-4374-25-4

**Published:** 2013-05-21

**Authors:** Pratap Karki, Eunsook Lee, Michael Aschner

**Affiliations:** 1 Department of Physiology, Meharry Medical College, Nashville, TN, USA; 2 Department of Pediatrics, Vanderbilt University Medical Center, 2215-B Garland Avenue, 11415 MRB IV, Nashville, TN, 37232-0414, USA

**Keywords:** Manganese, Neurotoxicity, Astrocytes, Glutamate transporters

## Abstract

Manganese (Mn) is an essential element that is required in trace amount for normal growth, development as well maintenance of proper function and regulation of numerous cellular and biochemical reactions. Yet, excessive Mn brain accumulation upon chronic exposure to occupational or environmental sources of this metal may lead to a neurodegenerative disorder known as manganism, which shares similar symptoms with idiopathic Parkinson’s disease (PD). In recent years, Mn exposure has gained public health interest for two primary reasons: continuous increased usage of Mn in various industries, and experimental findings on its toxicity, linking it to a number of neurological disorders. Since the first report on manganism nearly two centuries ago, there have been substantial advances in the understanding of mechanisms associated with Mn-induced neurotoxicity. This review will briefly highlight various aspects of Mn neurotoxicity with a focus on the role of astrocytic glutamate transporters in triggering its pathophysiology.

## Review

### Mn is essential but needs to be in the balance

Mn is a ubiquitous element present naturally in the environment, including water and food. In the human body, Mn is found in all tissues where it is required for normal amino acid, lipid, protein and carbohydrate metabolism, and ATP generation. Mn also participates in blood clotting and sugar homeostasis, immune responsiveness, digestion, reproduction, and bone growth [1-3]. It is a critical component of numerous metalloenzymes, including Mn superoxide dismutase, arginase, phosphoenolpyruvate decarboxylase and glutamine synthetase [4-6], to name a few. Despite its requirement in multiple physiological processes, elevated levels of Mn trigger toxicity, particularly within the central nervous system (CNS), causing cognitive, psychiatric and motor abnormalities [7,8]. In humans, Mn deficiency is rare as it is abundant in diets, but in extenuating conditions it may contribute to developmental defects, including malformation of bones, altered macromolecular metabolism, reduced fertility, weakness and enhanced susceptibility to seizures [3,9,10]. Mn deficiency has also been shown to induce skeletal defects and impaired lipid metabolism [11,12].

### Sources of human exposure

Mn is naturally abundant in the Earth’s crust (0.1%) and soil (40–900 mg/kg) [13,14]. Mn is found as oxides, carbonates and silicates in minerals. The versatile chemical properties of Mn have expanded its industrial use in glass, ceramics, paint and adhesive industries, as well as in welding. The wide usage of Mn in a range of industries has led to global health concerns. Indeed, occupational exposures to Mn have been documented in multiple industries, including ferroalloy smelting, welding, mining, battery, glass and ceramics [15-19].

The primary source of Mn exposure in the general human population is from diet, which is estimated to contain 0.9-10 mg Mn per day [20]. Rice, grains and nuts are rich sources of Mn with excess of 30 mg/kg, while Mn content in tea is 0.4-1.3 mg/cup. Mn drinking water levels are in the range of 1–100 μg/L [21]. The high Mn content in infant formulas compared to human milk, has recently drawn public attention [22], along with Mn in parenteral nutrition. The latter is considered a risk factor for Mn-induced toxicity since the normal intestinal regulatory mechanisms are bypassed, rendering intravenously delivered Mn 100% bioavailable [23,24]. Mn in fumes, aerosols or suspended particulate matters is deposited in the upper and lower respiratory tract, with subsequent entry into the bloodstream. An Mn-containing gasoline anti-knock additives additive, methylcyclopentadienyl manganese tricarbonyl (MMT), has been introduced in several countries, representing another source of Mn exposure via inhalation [25,26]. Designer drugs, such as methcathinone hydrochloride (ephedrine), where potassium permanganate is used as an oxidant for the synthesis of the illicit drugs [27] has also been shown to cause neurodegenerative sequalae in drug abusers, consistent with PD-like symptomology.

### Mn absorption, transport and excretion

Diet represents the major source of Mn in humans. The gastrointestinal tract absorbs 1-5% of ingested Mn; 60-70% of inhaled Mn is exhaled by the lungs [28,29]. The uptake of Mn is tightly regulated and excess Mn is excreted through the bile [30]. Both active transport and passive diffusion mechanisms regulate oral Mn absorption and the process is governed by various factors, including dietary levels of Mn and other minerals as well as age [20,30,31]. Among other metals, iron (Fe) stores are particularly important given the inverse relationship between cellular Fe levels and Mn uptake, as evidenced by increased transport of orally administered Mn in states of Fe deficiency [32,33]. In blood, Mn^+2^ is predominantly (>99%) in the 2+ oxidation state and mainly bound to β-globulin and albumin. A small fraction of Mn^+3^ is complexed with transferrin [34,35]. Mn (in the 2+ oxidation state) transport across the blood–brain barrier (BBB) and cell membranes is facilitated by the divalent metal ion transporter 1 (DMT1), *N*-methyl-d-aspartate (NMDA) receptor channel and Zip8 [36-38], to name a few. Transferrin is the most efficient transport system for Mn in the 3+ oxidation state [39]. Mn is distributed throughout the brain and the highest Mn levels are found in the globus pallidus and other nuclei of the basal ganglia (striatum, substantia nigra) [40,41]. DMT1 and transferrin also regulate Mn uptake both in astrocytes and neurons [42,43]. Generally, the intracellular Mn concentration is higher in tissues rich in mitochondria and pigmentation. The highest Mn levels are noted in bone, liver, pancreas and kidney compared to other tissues [44].

## Mn neurotoxicity

### Mn in neurological disorders

Chronic inhalation of air-borne Mn particulates represents the major cause of human neurotoxicity, though there is growing number of reports on Mn toxicity resulting from consumption of Mn-adulterated drinking water [45,46]. Occupational exposures represent the predominant source of excessive Mn exposure [47]. Manganism, first described by Couper in 1837 [48] is a clinical disorder characterized by psychological and neurological abnormalities that shares multiple analogous symptoms with idiopathic PD [Reviewed in [49]]. The early symptoms of manganism include hallucinations, psychoses and various behavioral disturbances soon followed by postural instability, dystonia, bradykinesia and rigidity [50]. Despite their resemblance in clinical features, manganism is clinically distinguishable from PD [51]. Mn-induced neurotoxicity affects mainly the globus pallidus as well as the cortex and hypothalamus [52,53], distinct from the striatal changes associated with PD. Excessive CNS Mn levels may contribute in the pathogenesis of PD, causing loss of dopamine in the striatum, death of non-dopaminergic (DAergic) neurons in the globus pallidus, and damage to glutamatergic and GABAergic projections [54,55]. Mn has also been shown to increase fibril formation by α-synuclein along with its expression and aggregation [53,56,57]. While playing a major role in the etiology of PD [58], the precise role of α-synuclein in Mn-induced neurotoxicity has yet to be determined. A role for Mn has also been advanced in the etiology of Huntington’s disease, amyotrophic lateral sclerosis and Alzheimer’s disease [Reviewed in [49]].

### Mechanism of Mn Neurotoxicity

#### Mitochondrial dysfunction

Mitochondria serve as the primary storage site for intracellular Mn where it is taken up by the calcium uniporter [59]. Mn is also an important cofactor for various mitochondrial enzymes and, thus, the elevation in Mn levels in this organelle can directly interfere with oxidative phosphorylation. Mn inhibits the function of F1-ATPase and the formation of complex I of the electron transport chain, thereby interfering with cellular ATP synthesis [60,61].

#### Oxidative stress

Elevated intra-mitochondrial Mn levels trigger oxidative stress, generating the excessive reactive oxygen species (ROS), causing mitochondrial dysfunction [61,62]. The transition of Mn^+2^ to Mn^+3^ increases its pro-oxidant capacity [63]. Mn-induced oxidative stress leads to the opening of mitochondrial transition pore (MTP), resulting in increased solubility to protons, ions and solutes, loss of the mitochondrial inner membrane potential, impairment of oxidative phosphorylation and ATP synthesis and mitochondrial swelling [64,65]. Furthermore, Mn exposure has also been linked to the activation of signaling pathways involved in response to oxidative stress, including nuclear factor kappa B (NF-_k_B) and activator protein-1 (AP-1) [66,67].

#### Apoptosis

Neuronal cell death by apoptosis has been considered to play a major role in neurodegenerative diseases, including PD [68]. Mn has been shown to trigger apoptosis in DAergic neurons in a caspase-3-dependent manner by activation of protein kinase C delta (PKC-δ) [69]. Similarly, Mn has been shown to cause apoptotic cell death in astrocytes by mitochondrial pathways involving cytochrome c release and caspase activation [64,70].

#### Inflammation

Although the oxidative stress induced by mitochondrial dysfunction is regarded as the major pathological mechanism of Mn neurotoxicity, recent studies also suggest a proinflammatory role for Mn, which involves the activation of glial cells characterized by the release of non-neuronal derived ROS, such as nitric oxide (NO), cytokines, prostaglandins and H_2_O_2_[71-73]. Mn potentiates the release of several cytokines, including TNF-α, IL-6, IL-1β from the activated glial cells, thereby activating various transcription factors including NF-_k_B, AP-1 and kinases including ERK, JNK, AKT, and PKC-α [Reviewed in [74]].

## The effect of Mn on astrocytic glutamate transporters

Astrocytes are the site of early dysfunction and damage in Mn neurotoxicity. Astrocytes are the most abundant CNS cells (~50% by volume), and they perform numerous essential functions for normal neuronal activity, such as glutamate uptake, glutamine release, K^+^ and H^+^ buffering and volume regulation [36,75,76]. Astrocytes accumulate up to 50-fold higher Mn concentrations compared to neurons, thus serving as the main homeostatic and storage site for this metal [75,77]. The intracellular concentration of Mn in astrocytes is ~50-75 μM where it is an essential cofactor for the astrocyte-specific enzyme glutamine synthetase, which catalyzes the conversion of glutamate to glutamine [78,79]. The excessive accumulation of Mn in astrocytes mediates neurotoxicity primarily by oxidative stress and impairment of glutamate transporters [80,81]. Mn toxicity has been shown to cause astrocytic alterations in primate models, and exposure of pathophysiologically relevant Mn concentrations in astrocytes *in vitro* causes time-and concentration-dependent cell swelling secondary to oxidative stress [82,83]. One of the proposed mechanisms of Mn-induced neurotoxicity in astrocytes is alteration in glutamate homeostasis due to impairment of glutamate transporters [84]. Mn has also been shown to downregulate the expression and function of glutamine transporters, resulting in reduced glutamine uptake [85]. The impairment of glutamate/glutamine transporters results in increased extracellular glutamate levels that elicit excitatory neurotoxicity. In support of this mechanism, we and others have shown that estrogen and selective estrogen receptor modulators protect astrocytes from Mn-induced neurotoxicity by upregulating the expression and function of glutamate transporters [86-88]. Moreover, Mn also activates selective cellular signaling pathways that mediate alterations in glutamate-glutamine homeostasis. The decrease in glutamine uptake after the activation of PKC-δ by Mn represents a typical example of the involvement of signaling pathways in Mn-induced neurotoxicity [89].

Although it is widely accepted that Mn impairs the expression and function of the two main glutamate transporters (GLAST and GLT-1), its mechanism of action at the transcriptional levels remains unknown. The increased production of ROS and TNF-α by Mn is thought to be the principal cause that leads to impairment in glutamate transporter function. ROS inhibit astrocyte glutamate uptake, and TNF-α decreases GLAST and GLT-1 protein and mRNA levels [90-92]. Oxidative stress also plays an important role in the regulation of glutamate transporter function since the activity of glutamate transporters is regulated by the redox state of reactive cysteine residues, with a dramatic decrease in activity once the reduced cysteine is oxidized [93]. Furthermore, glutamate uptake by the recombinant glutamate transporters EAAT1, EAAT2 and EAAT3 was found to be inhibited by peroxynitrite and H_2_O_2_ and restored upon treatment with the reducing agent, dithithreotol, suggesting a role for oxidative stress in the regulation of glutamate transporters activity [94]. TNF-α is a key neuroinflammatory mediator of neurotoxicity and neurodegeneration, and Mn increases the levels of this cytokine [95]. Several studies also corroborate the reduction in the expression and activity of glutamate transporters by TNF-α, highlighting its role as a negative regulator of the transporter [90,92]. NF-_k_B and MAPK signaling pathways mediate TNF-α-induced reduction in GLT-1 expression, since the inhibition of these pathways restores the decrease in TNF-α induced GLT-1 expression and function [92].

## Conclusion

Chronic excessive exposures to Mn represent a global health concern as growing evidences suggests that Mn accumulation in the brain may be a predisposing factor for several neurodegenerative diseases. Studies over the past several decades have provided invaluable insights into the cause, effects and mechanisms of Mn-induced neurotoxicity. The recent findings on the involvement of glutamate transporters and cellular signaling pathways in Mn-induced neurotoxicity provide not only new insights into the molecular mechanisms of Mn-induced neurotoxicity, but also provide new therapeutic targets in the development of novel drugs to attenuate the symptoms associated with manganism, PD and other related neurodegenerative disorders.

## Competing interests

The authors declare that there is no any competing interest.

## Authors’ contributions

PK drafted the initial manuscript, EL and MA corrected, edited and finalized it. All the authors read and approve the final version of the manuscript.
